# Adolescents Are More Utilitarian Than Adults in Group Moral Decision‐Making

**DOI:** 10.1002/pchj.821

**Published:** 2024-12-26

**Authors:** Yingying Jiang, Weiwei Zhang, Yingjia Wan, Michaela Gummerum, Liqi Zhu

**Affiliations:** ^1^ CAS Key Laboratory of Behavioral Science, Institute of Psychology Chinese Academy of Sciences Beijing China; ^2^ Department of Psychology University of Chinese Academy of Sciences Beijing China; ^3^ School of Child Development and Education China Women's University Beijing China; ^4^ Department of Psychology University of Warwick Coventry UK

**Keywords:** adolescent, group decision‐making, moral decision‐making, peer influence, utilitarian

## Abstract

This study explores how peers influence the moral decisions of Chinese adolescents (12‐ to 16‐year‐olds, *M*_age_ = 14.32, *n* = 84) and young adults (18‐ to 26‐year‐olds, *M*_age_ = 20.92, *n* = 99) in moral dilemmas. Participants were asked to make moral decisions individually and then collectively within groups of three to reach a consensus in Trolly dilemma and Footbridge dilemma. They were also required to evaluate the degree to which they felt their decisions were moral. Results showed that adolescents tended to choose “action” (pull the lever in Trolly dilemma, or push the man in Footbridge dilemma) more than adults, and evaluate their “no action” choice as more immoral than young adults across both individual and group settings. Adolescents showed consistent decision‐making patterns regardless of whether decisions were made individually or collectively, while adults were more likely to choose “no action” in group decision‐making. Our results suggest that adolescents are more utilitarian than young adults when making decisions in moral dilemmas, compared to young adults. Young adults are less likely to make utilitarian choices when they are in groups than when they make decisions individually.

## 1 Introduction

Imagine standing by railway tracks, where an empty trolley hurtles out of control toward five individuals working on the tracks. Adjacent to this track lies another, where only one person stands. Faced with the choice, would you pull a lever to redirect the trolley, sacrificing one life to save five? Dilemmas such as this Trolley dilemma have been used in moral philosophy and psychology to investigate what deliberations and decision processes might underlie people's choices in these dilemmas. The current study builds on this research in two important ways. First, it compares adults' decisions in these moral dilemmas with those of adolescents. Second, this study investigates whether groups' decisions in these dilemmas differ to those of individuals. Overall, this will allow us to further examine the influence of developmental and social factors on moral decision‐making and functioning.

### 1.1 Moral Dilemmas and Moral Decision‐Making

For decades, moral psychologists have used moral dilemmas to study moral decision‐making, moral reasoning, and other morality‐related processes across the lifespan. In his longitudinal and cross‐cultural studies, Kohlberg (1984) presented participants with moral dilemmas, defined as situations where two moral norms clash and an unequivocally “moral” solution is hard to decipher. One famous moral dilemma Kohlberg used is the Heinz dilemma where the moral norms of “not stealing” and “saving lives” clash. Kohlberg (1984) was interested in how participants decide in these dilemmas and, more importantly, how they reason about their choices. According to Kohlberg (1984), moral reasoning becomes increasingly complex throughout adolescence and into young adulthood and develops from focusing on outcomes, rewards, and punishments to paying attention to higher‐level considerations including law, social ethics, and conscience (Gibbs 2020). Based on this theory, adolescents may be more likely to focus on outcomes of actions in their moral reasoning than adults.

Moral dilemmas have also been used in philosophy and psychology as thought experiments to investigate whether people give more prominence to utilitarian or deontological considerations in their moral decisions. The Trolley and Footbridge dilemmas are two widely used moral dilemmas in these areas (Awad et al. 2020). In the Trolley dilemma, as we mentioned at the beginning, if the participant pulls a lever (action choice), the train can be diverted to another track where one worker is working. In the Footbridge dilemma, a runaway train is rushing toward five workers on the tracks. However, if the participant pushes a large person from a footbridge onto the tracks (action choice), the train can be stopped before killing the five workers. Thus, in both dilemmas, participants are asked to decide whether to take an action (i.e., pull the lever or push the man), thereby sacrificing one person to save five. According to utilitarian moral theories, an action that maximizes the utility for a greater number of persons should be considered moral (Mill 1863). In contrast, deontological moral theories emphasize universal moral principles or norms such as “do not harm/kill” (Kant 1948). As such, the “action” choice is commonly regarded as the indicating utilitarian, the “no action” choice deontological considerations.

A plethora of studies have used these dilemmas to investigate people's moral decisions in these dilemmas (Hauser et al. 2007). The Trolley and Footbridge dilemma have also been used to gain more general insights into the processes underlying people's moral decision‐making. The dual‐process model of moral decision‐making posits that this process involves both a cognitive system and an affective system. Choosing between “taking action” and “not acting” is understood as the outcome of the competition between these two systems (Conway & Gawronski 2013; Cushman 2013). “Taking action” typically results from the cognitive suppression of affective reactions. Affective prohibitions against interpersonal harm are activated by an aversion to the harmful act and are more likely to be associated with “not taking action” or the deontological choices (i.e., not pulling the lever or not pushing the man). Concern for the victim may serve as a significant developmental precursor to this aversion (Cushman 2013).

Fuzzy‐trace theory (FTT) was also proposed to explain how cognitive representations of moral dilemmas are essential in decision‐making. The core assumption of FTT is that individuals mentally represent information in two basic ways, the first is verbatim (the details literally) and the second is gist (simple bottom‐line meaning) (Reyna and Casillas 2009; Reyna and Panagiotopoulos 2020). Previous studies on adolescents' risky decision‐making suggested that adolescents who take risks tended to process verbatim details, while adults were more likely to rely on gist, ignoring trade‐offs and numerical magnitudes (Kwak et al. 2015; Reyna et al. 2011). In moral dilemmas such as Trolley dilemma or Footbridge dilemma, the background story will be summarized as gists such as “choosing whether to kill fewer people die to save more people” by adults, while adolescents might be more sensitive to the interference of numerical magnitudes.

Moreover, children usually develop their moral judgment through natural course of interactions with others (Kohlberg 1984). Previous research suggests that social experiences can shape individuals' moral judgments, particularly regarding concern for victims. For instance, school experiences may incline children and adolescents to prioritize the good of the majority, while broader social interactions may enable young adults to consider multiple perspectives on fairness, rather than solely apparent fairness (Hao and Liu 2016). Takagi (2014) coded adolescents' and young adults' moral reasoning in typical everyday‐life moral dilemmas and found age differences in reasons: adolescents (aged 14–16) were more likely to use the “deontological/rule” type of reasoning (concern for rules, social, or moral orders) than young adults aged 20–21, while young adults were more likely to employ the “deontological/fairness” (concern for fairness, rights, justice, respect, responsibility, trust etc.) than adolescents. This result is consistent with Gibbs' opinion that young adults pay more attention to higher‐level considerations (Gibbs 2020). However, to our knowledge, there has been limited investigation into age differences in moral decision‐making in the Trolley and Footbridge dilemmas and no study has investigated group versus individual decisions across development.

### 1.2 Moral Decision‐Making in Adolescents

In a recent study, participants from three age groups were asked to assess the acceptability of actions (or no actions) in the Trolley and Footbridge dilemmas (Bucciarelli 2015). Findings indicated that children, specifically 9‐ to 10‐year‐olds, exhibited a greater tendency toward utilitarian judgments compared to adult females. Moreover, adolescents, aged 13–14, also demonstrated a significantly higher inclination toward utilitarianism in their judgments compared to adult females. It is worth noting that the gender distribution in the study was not balanced.

Daniele and Bucciarelli (2016) used the same materials and procedure with 84 adults (equal numbers of males and females) and revealed that children reported taking an action (utilitarian choice) to be more acceptable than adults, but did not find a significant difference between adolescents and adults. Hao and Liu (2016) recruited 8‐ to 10‐year‐old children, 13‐ to 15‐year‐old adolescents, and younger adults from China to make decisions in the Trolley and Footbridge dilemmas and evaluate their decisions. Children and adolescents were more likely to choose “action” than adults, and the percentage of participants who judged taking actions as morally unacceptable increased with age. In sum, previous studies consistently found that children were more utilitarian than adults, but there were mixed findings when comparing the moral decisions of adolescents and adults.

Furthermore, culture influences individuals' moral judgment and decision‐making (McNamara et al. 2019). Awad et al. (2020) used three moral dilemmas, the Trolley dilemma, Footbridge dilemma, and the Loop dilemma where if the participant did nothing the trolley would hit five persons on the track, but if the participant chose to pull the level, the trolley would hit the one person and then ground to halt to save five persons on the main track. The authors analyzed responses from 70,000 participants in 10 languages and 42 countries and found that participants from Asian countries were more likely to reject the utilitarian choice than those from America and Europe (Awad et al. 2020). Rhim, Lee, and Lee (2020) made a cross‐cultural comparison of Korea and Canada and also found similar results that participants from Korea were less frequently to choose utilitarian choice. There were two possibilities that people in Asian culture are more intended to reject utilitarian choice. Researchers in moral decision‐making suggested that although moral emotions (such as guilt, shame, or regret) were universal across cultures, shame and guilt were more important in collective culture. People in East Asian countries reported experiencing moral emotions more frequently and intensely (Scollon et al. 2004; Tracy and Matsumoto 2008). Besides, utilitarian choice is found to be related to a low level of empathy concern (Gleichgerrcht and Young 2013), while people in collective cultures exhibit a higher level of empathy (Cheon et al. 2011; Luo et al. 2015). However, there is limited research on moral decision‐making among adolescents in underrepresented cultures other than the WEIRD (Western, Educated, Industrialized, Rich, and Democratic) populations. The current study will assess the moral decision‐making of adolescents and adults from China.

### 1.3 Moral Decision‐Making in Groups

People often make decisions in groups. However, group decision‐making and the dynamic process of social influence on moral decisions seem to have been neglected by researchers (De Cremer and Moore 2020). Most studies on moral dilemmas have been conducted at the individual level, and studies on how groups make decisions in moral dilemmas are limited (Lehnert, Park, and Singh 2015). People often have different moral opinions about the same moral issue. Group decision‐making integrates and considers individual information (Haller et al. 2018). Studying group decision‐making in moral dilemmas is also theoretically interesting.

Recently, Curşeu et al. (2020) used 10 moral dilemmas and found that group‐level utilitarianism was higher than the average individual utilitarianism, and group‐level utilitarianism could be predicted by individual‐level utilitarianism. Keshmirian, Deroy, and Bahrami (2022) used eight sacrificial dilemmas including the Footbridge dilemma and also found that group judgments in moral dilemmas were more utilitarian than individual judgments. Nonetheless, the participants in the aforementioned studies were adults, leaving us uncertain about whether adolescents may make different moral decisions in peer groups.

A large proportion of adolescents' decisions are made in peer groups (Knoll et al. 2015). Takezawa et al. (2006; Gummerum et al., 2008) used economic distribution games, namely the ultimatum game (UG) and dictator game (DG), and found that in groups of 13‐year‐old adolescents pro‐social and fair peers were more influential in the final group decision whereas in groups of 11‐year‐olds selfish peers were more influential. Ahmed et al. (2020) found that 11‐ to 18‐ year‐old (male) adolescents were less influenced by peers' pro‐social and antisocial behavior than younger children. Chierchia, Piera Pi‐Sunyer, and Blakemore (2020) recruited 11‐ to 14‐year‐olds, 15‐ to 18‐year‐olds, and 23‐ to 25‐year‐olds to participate in a donation task, and they found similar trends. Zhu et al. (2008) recruited 8‐ to 9‐year‐olds, 11‐ to 12‐year‐olds, 13‐ to 14‐year‐olds, and 18‐ to 19‐year‐olds in DG and UG tasks in China, and found that there was no significant difference between individual decisions and group decisions. They proposed that these results might be due to the strategy in the group decision‐making process in that participants tended to take the average value of individual decisions, so the difference between the mean of individual decisions and group decisions was not significant. Decision‐making in moral dilemmas primarily hinges on individuals' moral judgment and moral reasoning than on the pro‐social behavior in UG or DG, and the strategy of average value was no longer applicable in moral dilemmas. Since adolescents' decision‐making would be more influenced by peers, it is reasonable to predict that the difference between individual and group conditions would be larger in adolescents than in adults.

## 2 Current Study

This study aims to investigate individual and group moral decision‐making among adolescents and young adults, employing the Trolley and Footbridge dilemmas. Participants initially made individual and private decisions in both dilemmas, followed by group discussions in groups of three aimed at reaching a consensus. We anticipate that in individual decisions adolescents will exhibit a higher inclination towards “action” choices compared to adults. Concerning group decisions, we also expect that the differences between individual and group decisions would be larger in adolescents. Regarding moral ratings, since school experiences may teach children and adolescents to prioritize the good of the majority, and broader social interactions may enable young adults to consider multiple perspectives on fairness, rather than solely apparent fairness (Hao and Liu 2016), we hypothesize that adolescents would consider “action” as more moral than adults, while adults may believe “no action” to be more moral.

### 2.1 Method

#### 2.1.1 Participants

Previous studies did not study fixed group sizes. For example, Keshmirian, Deroy, and Bahrami (2022) recruited 73 participants in 16 mixed‐gender groups, each including four or five members, and Curşeu et al. (2020) included 221 students in 67 groups and group size ranged from 2 to 6. We tried to keep the impact of group size, age, and gender constant. A total of 183 participants (61 groups of three, matched in age and gender) were recruited online, including 84 adolescents (48 females) ranging from 12 to 16 years (*M*_age_ = 14.32, *SD* = 0.48) and 99 young adults (48 females) ranging from 18 to 26 years (*M*_age_ = 20.92, *SD* = 1.63). Adolescents were from one middle school in a city in Northern China, and young adults were college and graduate students from a variety of universities in Northern and Central China. All the young adults did not begin their jobs when they participated in this study. They were asked to invite two other peers of the same age and gender to participate online together. Thus, group members knew each other. We did not collect participants' socioeconomic status information, but given the demographic information of the region they live in, we believe most participants came from middle‐income families. Informed consent was obtained from each adult participant and adolescents' parents. This research was approved by the Institutional Review Board (IRB) of Institute of Psychology, Chinese Academy of Sciences.

#### 2.1.2 Design and Procedure

Our study employed a 2 (age group: adolescents vs. adults) × 2 (condition: individual decision‐making vs. group decision‐making) design. Each participant participated in both decision‐making conditions. The dependent variables were moral choices and moral ratings. Trolley dilemma and footbridge dilemma were adopted from Bucciarelli (2015). Participants were instructed to decide what to do in each dilemma (i.e., pull a lever or not to pull in trolley dilemma; push a man or not to push in footbridge dilemma) and evaluate whether their decision was moral from 1 (*totally immoral*) to 7 (*totally moral*).

The experiment was conducted online via Tencent Meeting (meeting.tencent.com) due to the COVID‐19 pandemic. Participants were invited into the Tencent Meeting session in groups of three members. General instructions were presented to them orally, and then each participant received an internet link of the experiment. The experiment was programmed through online software (www.wjx.cn), and participants engaged with the tasks in a fixed order. After filling out a consent form, participants were first asked to read and respond individually to the moral dilemmas, which were presented in a counterbalanced order. The individual condition was finished after participants submitted their individual responses. Then, in the group condition, the three participants were asked to discuss and then arrive at a unanimous group decision. During the discussion, the experimenter left the online meeting room and came back when group members finished their discussion. There was no time limit, but most participants provided their responses within 5 min in each scenario. At the end of the study, participants were thanked and received a certain amount of compensation.

#### 2.1.3 Coding and Reliability

Open‐ended questions about why groups made their specific decision were asked in the group condition after participants reached an agreement. The coding scheme was developed from 50% of the answers, randomly selected across age groups and dilemmas. Participants chose “action” mainly because they supported sacrificing one person's life could save five persons (i.e., for utilitarian reasons). Therefore, we coded reasons given by groups who chose “no action” in each dilemma in more detail. Reasons for “no action” were coded into six dimensions: (1) “Legal consequence” referred to the legal liabilities that choosing to act may bring upon oneself (e.g., “Killing that person means you'll face the social and legal trouble that comes with it.”); (2) “Accountability” meant that participants believed the death risk five persons on the railway were facing was due to their own behaviors (e.g., “It's the fault of those five individuals. They shouldn't stand on the tracks.”); (3) “Equal right to life” meant that participants believed human life cannot be sacrificed to save others simply because there were fewer of them (e.g., “Everyone has the right to life; Every life is precious and cannot be compared by numbers.”); (4) “Moral principle” referred to the principle in morality such as not causing harm to others (e.g., “No one has the right to decide the life or death of another person.”); (5) “Other reasons” referred to less relevant reasons that were difficult to categorize into the previous types (e.g., “Maybe those five people just want to commit suicide; Even if we push, we can't save five persons. We can't move the fat man”); (6) “Didn't mention any reasons” always referred to the groups where the participants were all in agreement so they did not report reasons.

Multiple categories were permitted. A total of 50% of the answers (29 groups) were randomly selected to be coded by two independent coders and the inter‐coder agreement was calculated as Cohen's kappa was 0.85. Discrepancies between two coders were discussed subsequently.

### 2.2 Results

#### 2.2.1 Trolley Dilemma

##### 2.2.1.1 Differences in Moral Decision‐Making Across Age and Conditions

Chi‐square tests showed that adolescents tended to choose the utilitarian response “take action” in both individual and group conditions more often than the deontological “no action” response (individual: *χ* ^2^(1) = 25.19, *p* < 0.001; group: *χ* ^2^(1) = 37.39, *p* < 0.001, Table 1). Conversely, adults had no preference in the individual condition (*χ* ^2^(1) = 0.82, *p* = 0.366) and were more likely to choose “no action” in the group condition (*χ* ^2^(1) = 7.84, *p* = 0.005, Table 1).

**TABLE 1:** Descriptive results of participants choosing to “act” in each condition in Trolley dilemma (*n* [%]).

	Individual	Group
Adolescents	65 (77.4%)	66 (84.6%)
Adults	45 (45.5%)	33 (35.5%)

**FIGURE 1:**
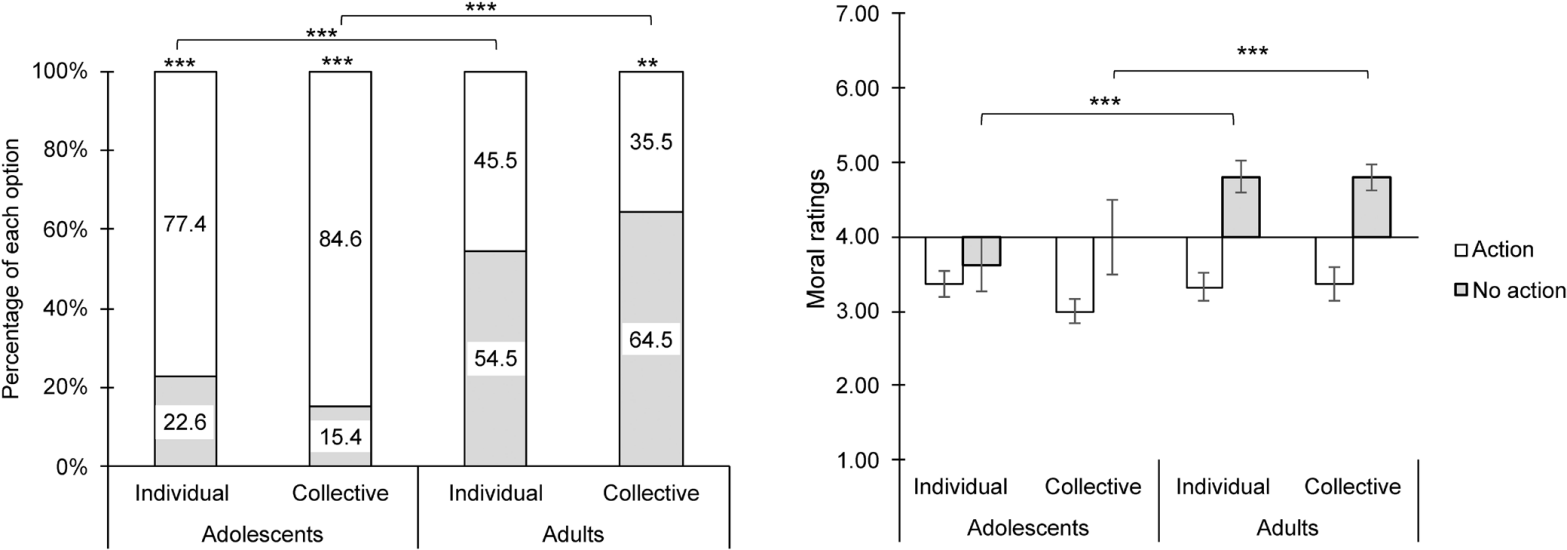
Moral decisions (A) and moral ratings (B) on the trolley dilemma in each age group and decision‐making conditions. Significant differences are marked with asterisks (**p < 0.01; ***p < 0.001).

A 2 (age group: adolescents vs. adults) × 2 (condition: individual vs. group) repeated measures logistic regression analysis revealed a significant main effect of age group, *χ* ^2^(1) = 40.42, *p* < 0.001 and a significant interaction effect of age group × condition, *χ* ^2^(1) = 4.81, *p* = 0.028. There was no main effect of condition, *χ* ^2^(1) = 0.01, *p* = 0.946. Further paired comparisons showed that adolescents were more likely to choose “action” than adults in both individual and group conditions (*p* < 0.001). Adolescents chose similarly in individual and group conditions, whereas adults tended to be more likely to choose “no action” in the group condition (*p* = 0.073, Figure 1A).

##### 2.2.1.2 Differences in Moral Evaluation Across Age and Conditions

A Generalized linear model test was conducted under each moral choice. Moral rating was the dependent variable. Age group, context (individual vs. group), and their interaction were independent variables. For participants who chose “action,” there were no significant results either in main effects or interaction effect. However, for participants who chose “no action,” the analysis revealed a significant main effect of age group, *χ* ^2^(1) = 7.67, *p* = 0.006 (Table 2). There was no main effect of condition or interaction effect. Further paired comparison in the main effect of age group showed that adolescents' ratings were lower than adults when they chose “no action” (*p* < 0.001) (Table 2, Figure 1B).

**TABLE 2:** The descriptive results of participants' moral ratings in each condition in Trolley dilemma (M [SD]).

	Individual		Group	
	Action	No action	Action	No action
Adolescents	3.37 (1.45)	3.63 (1.64)	3.00 (1.36)	4.00 (1.81)
Adults	3.33 (1.35)	4.81 (1.54)	3.36 (1.32)	4.80 (1.41)

##### 2.2.1.3 Changes in Choices and Ratings Between Individual and Group Conditions

**FIGURE 2:**
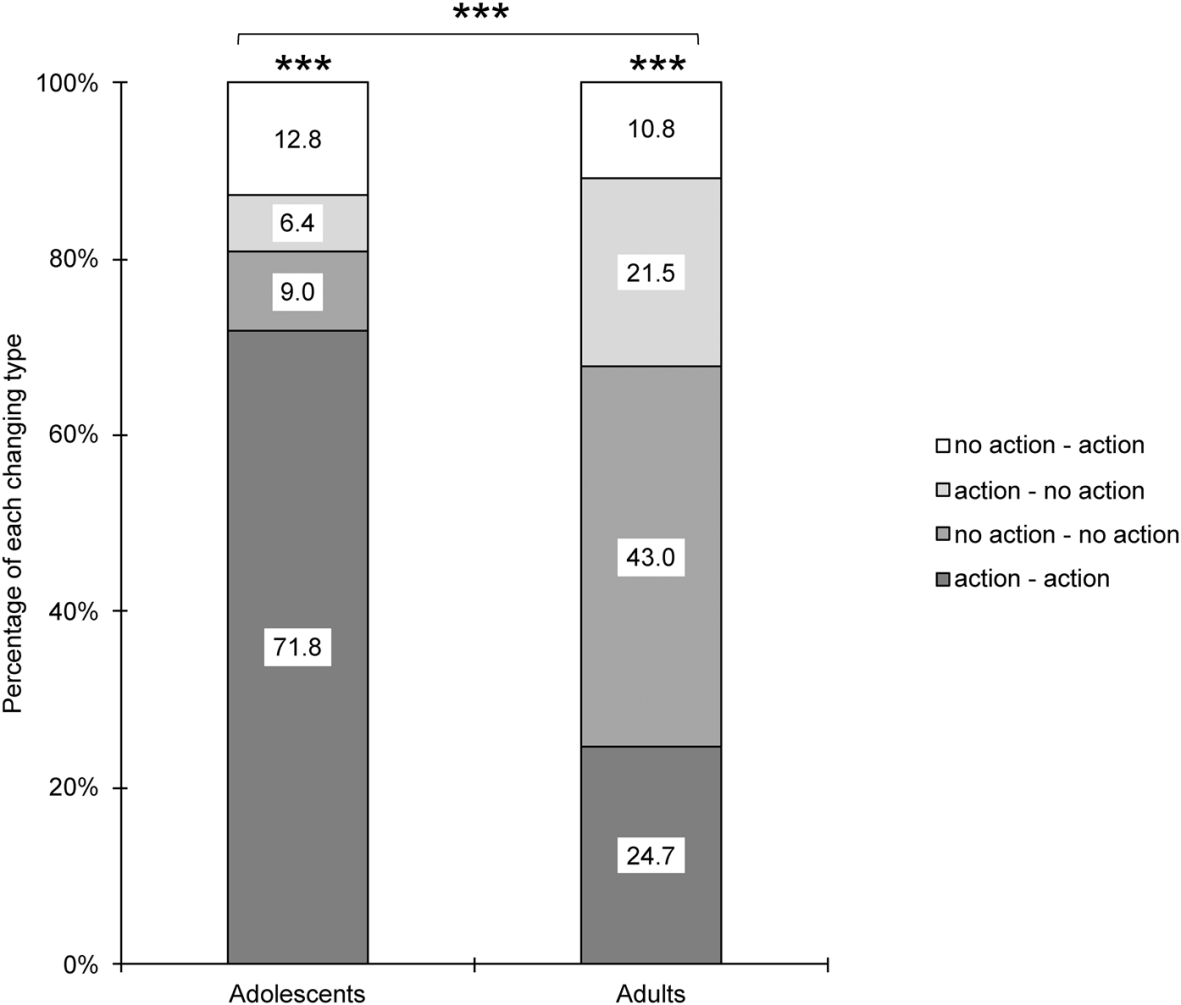
The percentage of choice changes from individual decision‐making condition to group decision‐making condition in the trolley dilemma. Significant differences are marked with asterisks (***p < 0.001).

To further analyze the difference in moral decisions between individual and group conditions in the trolley dilemma, we coded participants' changes in moral decisions. Four such choice change types were created: choosing “action” individually and agreeing to “action” in group decisions (action–action); choosing “no action” and agreeing to “no action” in group decisions (no action–no action); choosing “action” individually and agreeing to “no action” in group condition (action–no action); choosing “no action” individually and agreeing to “action” in group condition (no action–action). The percentages of these four types differed in each age group (*p* < 0.001), and the age differences were also significant, *χ* ^2^(3) = 44.99, *p* < 0.001, Cramer's *V* = 0.513. About 71.8% of adolescents chose “action” individually and agreed to “action” in group decisions, while the percentage of type “action–no action” in adults (21.5%) was higher than in adolescents (6.4%) (Figure 2).

We also analyzed changes in moral evaluations associated with the different choice change types. The moral evaluation change ratings were calculated by subtracting the ratings in the individual condition from the group condition. The moral evaluation change rating in each choice change type was compared with 0 using one‐sample Wilcoxon signed rank test. If the moral evaluation change rating was significantly higher than 0, this would indicate that participants raised their moral rating significantly in the group condition than individual condition. We found that adults raised their moral ratings in the “action–no action” type (*M*_change_ = 1.70, *p* = 0.005, Table 3) and decreased their moral ratings in the “no action–action” type (*M*_change_ = −1.80, *p* = 0.010, Table 3). That is, when adults changed their choice from “action” in the individual condition to “no action” in the group condition, they believed this change was more moral; When adults who chose “no action” individually changed their choice in the group condition to “action,” they rated their choice more immoral in group condition. Moreover, we also found there was a trend that adolescents in “action–action” type decreased their morality rating in the group condition (*M*_change_ = −0.25, *p* = 073, Table 3).

**TABLE 3:** The descriptive results of participants' changes in moral ratings within each change type in Trolley dilemma.

		*M*	SD	*p*	*n (%)*
Adolescents	Action‐action	−0.25	1.64	0.073	56 (71.8%)
	No action–no action	0.57	2.57	0.854	7 (9.0%)
	Action–no action	−0.60	0.89	0.180	5 (6.4%)
	No action–action	−0.70	2.54	0.382	10 (12.8%)
Adults	Action–action	−0.09	1.20	0.785	23 (24.7%)
	No action–no action	0.00	1.68	0.953	40 (43.0%)
	Action–no action	1.70	2.23	0.005	20 (21.5%)
	No action–action	−1.80	1.14	0.010	10 (10.8%)

#### 2.2.2 Footbridge Dilemma

##### 2.2.2.1 Differences in Moral Decision‐Making Across Age and Conditions

Chi‐square tests showed that adolescents had no preference in individual condition (*χ* ^2^(1) = 0.76, *p* = 0.383) and tended to choose “action” in the group condition over “no action” with marginal significance (*χ* ^2^(1) = 3.86, *p* = 0.050, Table 4). Adults tended to choose “no action” in both individual and group conditions (individual: *χ* ^2^(1) = 26.27, *p* < 0.001; collective: *χ* ^2^(1) = 87.36, *p* < 0.001, Table 4).

**TABLE 4:** Descriptive results of participants choosing to “act” in each condition in Footbridge dilemma (*n* [%]).

	Individual	Group
Adolescents	46 (54.8%)	51 (60.7%)
Adults	24 (24.2%)	3 (3.03%)

**FIGURE 3:**
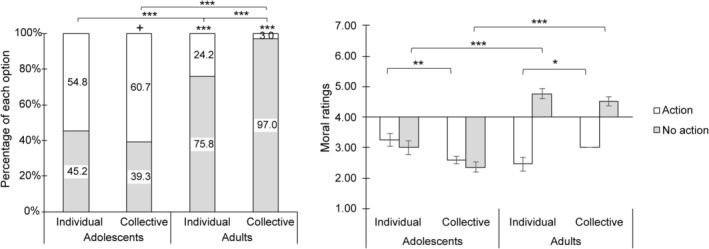
Moral decisions (A) and moral ratings (B) on the footbridge dilemma in each age group and decision‐making conditions. Significant differences are marked with asterisks (+*p* < 0.08; *p < 0.05; **p < 0.01; ***p < 0.001).

A 2 (age group: adolescents vs. adults) × 2 (condition: individual vs. group) repeated measures logistic regression analysis revealed a significant main effect of age group, *χ* ^2^(1) = 47.29, *p* < 0.001, a main effect of condition, *χ* ^2^(1) = 10.45, *p* = 0.001, and an interaction effect, *χ* ^2^(1) = 15.94, *p* < 0.001. Further paired comparisons showed that adolescents were more likely to choose “action” than adults in both individual and group conditions (*p* < 0.001). Adolescents chose similarly in both individual and group conditions (*p* = 0.333), while adults were more likely to choose “no action” in the group than the individual condition (*p* < 0.001, Figure 3A).

##### 2.2.2.2 Differences in Moral Evaluation Across Age and Conditions

Generalized linear model test was conducted for each moral choice. For participants who chose “action,” analysis revealed an interaction of age group and condition, *χ* ^2^(1) = 15.94, *p* < 0.001. Further paired comparison of an age group × condition interaction effect showed that adolescents' ratings declined significantly in the collective condition (*p* = 0.001, Table 5), while adults' ratings increased in collective conditions (*p* = 0.014) (Table 5, Figure 3B). However, for participants who chose “no action,” the analysis revealed a significant main effect of age group, *χ* ^2^(1) = 106.62, *p* < 0.001, and a significant main effect of condition, *χ* ^2^(1) = 5.55, *p* = 0.011. Further paired comparison in the main effect of age group showed that adolescents' ratings were lower than adults (Table 5, *p* < 0.001), and paired comparison in the main effect of condition showed that ratings in the collective condition were lower than individual condition (*p* < 0.001) (Table 5, Figure 3B).

**TABLE 5:** The descriptive results of participants' moral ratings in each condition in Footbridge dilemma (M [SD]).

	Individual		Group	
	Action	No action	Action	No action
Adolescents	3.24 (1.43)	3.00 (1.40)	2.59 (0.78)	2.36 (0.99)
Adults	2.46 (1.10)	4.77 (1,51)	3.00 (0.00)	4.53 (1.49)

##### 2.2.2.3 Changes in Choices and Ratings Between Conditions

**FIGURE 4:**
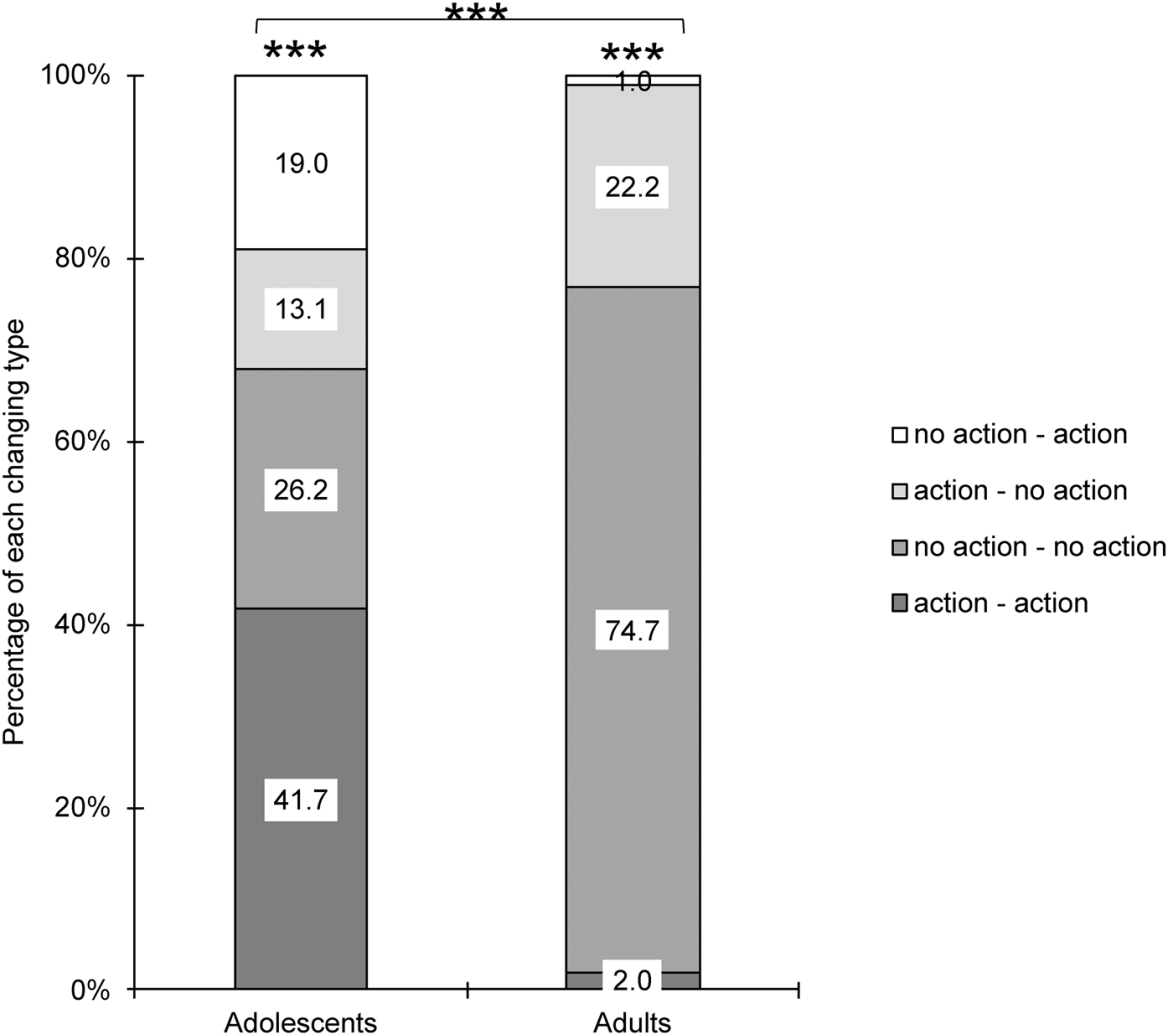
The percentage of choice changes from individual decision‐making condition to group decision‐making condition in the footbridge dilemma. Significant differences are marked with asterisks (***p < 0.001).

The same four types of patterns for participants' choices in the individual and group conditions as for the trolley dilemma were created. The four choice patterns differed in percentage in each age group (*p* < 0.001), and age differences were significant, *χ* ^2^(3) = 73.77, *p* < 0.001, Cramer's *V* = 0.635. About 74.7% of adults chose “no action” individually and agreed to “no action” in group decisions, while the percentage of “action–no action” in adults (22.2%) was higher than in adolescents (13.3%) (Figure 4).

We also analyzed changes in moral evaluations associated with the different choice change types in line with the analyses conducted for the Trolley Dilemma. Adults in the “action–no action” type raised their moral ratings (*M*_change_ = 1.64, *p* = 0.002, Table 6), while adolescents' ratings decreased significantly (*M*_change_ = −1.64, *p* = 0.014, Table 6). There was also a significant difference in moral rating changes between age groups in the “action–no action” type, that is, the changes in rating in adults were higher than in adolescents. Moreover, we also found there was a trend that adolescents in “action–action” type decreased their morality ratings in the group condition (*M*_change_ = −0.49, *p* = 0.051, Table 6).

**TABLE 6:** The descriptive results of participants' changes in moral ratings within each change type in Footbridge dilemma.

		*M*	SD	*p*	*n* (*%*)
Adolescents	Action–action	−0.49	1.38	0.051	35 (41.7%)
	No action–no action	−0.50	1.85	0.239	22 (26.2%)
	Action–no action	−1.64	1.57	0.014	11 (13.1%)
	No action–action	−0.44	1.79	0.320	16 (19.0%)
Adults	Action–action	0.00	0.00	1.000	2 (2.0%)
	No action–no action	−0.07	1.35	0.716	74 (74.7%)
	Action–no action	1.64	1.97	0.002	22 (22.2%)
	No action–action	−4.00	—	0.317	1 (1.0%)

##### 2.2.2.4 Reasons in Group Decision‐Making Condition

Open‐ended questions about why groups made their specific decision were asked in the group condition after participants reached an agreement. Participants chose “action” mainly for utilitarian reasons, so we coded reasons given by groups who chose “no action” in each dilemma in more detail in Table 7. Fisher's exact test was used to analyze the differences. The reasons between age group were not significantly different, *χ* ^*2*^_TD_(5) = 4.68, *p* = 0.370, *χ* ^2^_FD_(5) = 2.87, *p* = 0.772. However, Chi square goodness of fit test showed there was a significant difference within reasons in adult groups, *χ* ^*2*^_TD_(5) = 11.35, *p* = 0.045, *χ* ^2^_FD_(5) = 15.83, *p* = 0.007. Adults paid more attention to accountability about the scenario than other reasons.

**TABLE 7:** Reasons in group decision‐making for each moral dilemma.

Reasons	Trolley dilemma		Footbridge dilemma	
	Adolescents	Adults	Adolescents	Adults
Legal consequence	0	8 (27.6%)	5 (31.25%)	10 (21.74%)
Accountability about the scenarios	2 (40%)	10 (34.5%)	3 (18.75%)	16 (34.78%)
Equal right to life	0	2 (6.90%)	1 (6.25%)	4 (8.70%)
Moral principle	0	3 (10.34%)	0	2 (4.35%)
Others	1 (20%)	3 (10.34%)	3 (18.75%)	7 (15.22%)
Did not mention the reason	2 (40%)	3 (10.34%)	4 (25%)	7 (15.22%)

## 3 Discussion

This study explored individual and group decision‐making in moral dilemmas among adolescents and adults. Results found that, when making individual decisions, adolescents were more likely to choose the utilitarian “action” choice than adults do. Adolescents were also more inclined to evaluate the deontological “no action” choice as more immoral than young adults in both individual and collective conditions. Adolescents acted similarly in both individual and group decisions, while adults were more likely to choose “no action” in group decision‐making. Adolescents' ratings for their moral decisions were lower in the group condition than in the individual condition no matter what they chose, while adults' ratings did not differ in individual and group conditions. Adult groups paid more attention to accountability about the scenario than other reasons.

### 3.1 Age Differences in Moral Decision‐Making in Dilemmas

We found that adolescents were more likely to sacrifice one person's life to save more lives than adults in both individual and collective conditions and thus showed more utilitarian responses. The age difference in the footbridge dilemma is consistent with the results of Hao and Liu (2016). Fuzzy‐trace theory predicts developmental reversals in reasoning within situations characterized by uncertainty and risk. According to this theory, adolescents tend to engage in more calculated reasoning and are less inclined to process the gist of the information (Brainerd & Reyna 2015). There may be consistent developmental patterns in moral decision‐making, with adolescents more likely adopting utilitarian approaches in moral dilemmas (calculating the costs and benefits of their actions for the greatest number of people), while adults are guided more by general values and norms, such as not causing harm. Another possible explanation could be attributed to the influence of school experiences, where Chinese adolescents may place greater emphasis on the well‐being of the majority, particularly due to the emphasis on concepts like “unity and friendship” within Chinese educational settings for children and adolescents. Moreover, broader social interactions enable young adults have a more multi‐dimensional concept of fairness and pay more attention to a higher‐level concern for rights and responsibility. More knowledge about the consequence of individuals will evoke more negative moral emotions (such as guilty, shame, or regret) before deciding to harm others in both moral dilemmas, which may suppress the cognitive compare the numerical magnitude.

It is worth noting that an intriguing pattern emerged in our study. When choosing the utilitarian “action” option, both adolescents and adults tended to evaluate their choice as immoral. When choosing the deontological “no action,” adolescents still considered it immoral, whereas adults tended to think it was moral. In the process of decision‐making, adolescents in our studies held unstable moral judgment and realized each choice had its rationality and death consequence: The “action” choice saved more persons but the participants killed an innocent person, while the “no action” choice did not involve personal harm but the participants chose to stand by witnessing more people dying. Adolescents felt guilty or regret would rate their actions as morally less acceptable. Besides, young adults tended to provide various justification when choosing not to act and they were more tended to believe the “accountability about the scenario”. That is, adults were more likely to think that the death risk five persons on the railway were facing was due to their own behaviors. Their justifications might explain the age difference in morality ratings: adults used reasons to justify “no action” that could be seen as more self‐interested, and accountability reasons are the type of justifications often used when trying to rationalize (im) moral decisions, such as blaming the victims (see Bandura et al. 2001; Keller 1984).

Our study was not cross‐cultrual, but we still try to compare our results with previous related studies. Awad et al. (2020) compared results in three moral dilemmas from 70,000 participants in 42 countries and found about 32% of Chinese young adults chose to sacrifice one person to save five in the footbridge dilemma. Our result in young adults was consistent with the results in the larger sample in their study. Upon further comparison, it became evident that Chinese adolescents (55%) were more inclined to choose the “action” option compared to Italian adolescents (36%), whereas the percentages of adults in both studies were approximately around 30% (Daniele and Bucciarelli 2016). This results did not match the prediction that people in collective culture are more likely to reject to act. Since this analysis was not direct, these results should be interpreted cautiously. But this results still suggest the cultural difference may change at each age stage and a larger matched sample is needed for further cultural compare. A recent study about moral judgments in trolley dilemma in cultural diverse sample in Eastern, Western and Southern countries found that the personal force (i.e., whether or not to use personal effort to kill the one person) effect was universal in all cultural clusters, but found no strong association between collective/individual culture and moral judgments (Bago et al. 2022).

### 3.2 Differences Between Individual and Group Moral Decision‐Making

Adolescents made similar decisions in both individual and group conditions, while adults were less likely to choose the utilitarian option in the group. This result did not support our hypothesis that the differences between individual and group decisions would be larger in adolescents. Most previous studies found adolescents were more likely to be influenced by peers in other pro‐social and antisocial tasks. Our findings suggest that age‐related disparities in peer influence may vary depending on the specific task, as noted by Smith et al. (2014). Moreover, it is important to recognize that different forms of moral or pro‐social behaviors may have their own distinct correlates and may be influenced by the unique characteristics of the task, as highlighted by Carlo and Padilla‐Walker (2020). While the pro‐social tasks of Dictator Game, Ultimatum Game, and donations measure moral behaviors such as sharing, moral dilemmas measure moral judgment in more complex and significant situations that involve people's lives. When further analyzing the changes of choices between conditions, we found that the result of adolescents' similar decisions in both conditions was mainly because comparable changes from “action” to “no action”, others change from “no action” to “action”. That is, there were simultaneously two changes in opposite directions among adolescents, whereas in the adult participants, the main change was from “action” to “no action”. This changing pattern indicates that adolescents were also influenced by peers' choices in one group. They may pay attention to the rationality behind the two choices and change their decisions into opposite directions. At the same time, although young adults were uncertain whether to act individually, but they were more likely to change to “no action.” This change was not due to the majority choice, since in trolley dilemma the majority choice in adolescents was “action” and in Footbridge dilemma adolescents had similar percentage in two choices, while the adults had no bias in Trolley dilemma and were more likely to choose “no action” in Footbridge dilemma. The changing pattern may due to unstable moral judgment in adolescents and more social experience in adults.

It is worth noting that our findings among adults differ from those of a previous study, which reported that adults were more inclined to support taking action as a group (Keshmirian, Deroy, and Bahrami 2022). This difference may be due to a different experimental setting: Keshmirian et al. asked the participants from a third‐person perspective, while we asked participants to make a moral decision from the first‐person perspective. Researchers often utilize the third‐person in moral dilemmas to evaluate moral judgment, while using the first‐person to ask the participants to make moral decisions in the perspectives of the protagonist in the dilemmas. However, moral judgment is not always consistent with moral decisions (Gawronski et al. 2017). Moral judgments are mainly made from third‐person perspective and may rely more on normative prescriptions and beliefs (Nichols and Mallon 2006), while moral decisions are made by taking personal experiences and motivations into greater account (Zeelenberg et al. 2008). Adults, having more life experience, were more aware of the potential consequences of their actions. This awareness often led them to choose “no action” and avoid intervening in group decision‐making situations, particularly when responsibility was shared among group members.

In terms of moral ratings, it is noteworthy that adolescents assigned lower morality ratings to their decisions in the group condition compared to those in the individual condition. However, there was no significant discernible difference in moral ratings among adults across the two conditions. Irrespective of whether adolescents stuck to their choice of “action” or changed to “no action” in the group condition, their moral rating decreased. However, adults believed that their changes from “action” to “no action” in groups were moral and their changes from “no action” to “action” were immoral. When individuals have a discussion, they learn about each option's justification in moral dilemmas (Hao and Liu 2016). After discussion, adolescents might have been more likely to realize that each option will cause death and thus evaluate their choices as more immoral. Adults may have raised their ratings when they changed from “action” to “no action” for two reasons: This change is in line with their emphasis on accountability about the scenario. Moreover, group discussion increases the degree of rationality and the consideration of self‐interest. When they chose not to intervene in the scenarios in groups, they tended to approach their choices with a focus on avoiding potential troubles, viewing “no action” as a morally preferable option.

## 4 Limitations and Future Directions

Our experiment was conducted online as a response to the COVID‐19 pandemic, and it is important to acknowledge that there may be differences between online and face‐to‐face discussions that could potentially impact the results. For example, research has shown that social conformity on moral issues differs between contexts that are high in social presence (e.g., other group members are in the same room) and contexts low in social presence (e.g., groups interacting online). For future studies, it would be valuable to compare group decision‐making in moral dilemmas in contexts that are high and low in social presence. It should be noted, however, that making group decisions online is now a common feature of school and work environments, especially since the COVID‐19 pandemic.

Although we found that most reasons could be included into deontological principles in adolescents and adults, our analysis of the reasons participants gave for “no action” shows that this cannot always be interpreted as deontological decision (see also Gawronski et al. 2017). Future studies could utilize other paradigms to distinguish different motivations underlying moral dilemmas, such as CNI model, which is a multinomial model that allows researchers to quantify sensitivity to consequences (C), sensitivity to moral norms (N), and general preference for inaction versus action irrespective of consequences and norms (I) in responses to moral dilemmas (Gawronski et al. 2020).

The reasons in moral dilemmas are valuable to analyze the process of moral decision‐making. We coded reasons in group condition and found age group differences in reason dimensions. Future studies can collect both reasons in individual and group condition to investigate whether the reason dimension will change in different decision‐making condition. Moreover, adolescents are more likely to choose to act in these moral dilemmas for the same utilitarian reason, but may have diverse reasons when choose not to act, which needs a large sample to ensure there is sufficient data for further coding and analyze.

Future studies can further explore the differences between individual and group conditions and the mechanism underlying the differences. Previous studies divided the strategies in group decision‐making into two types: normative and informational strategy. These types differed in whether individuals sincerely change their actual private opinion after group discussion. Researchers found that adults' group discussion only changed their collective decisions, but did not affect their private decisions after group discussion (Keshmirian, Deroy, and Bahrami 2022). Future studies could test these two strategies with adolescents. Participants' individual differences are also valuable when analyzing the mechanism, for instance, socio‐economic status may be an important factor that influences individuals' moral judgment, which was not measured directly in our study.

We investigated individual and group moral decision‐making among adolescents and young adults, employing the Trolley dilemma and Footbridge dilemma. This study found that adolescents tended to choose “action” more than adults, and evaluate their “no action” choice as more immoral than young adults across both individual and collective settings. Adolescents showed consistent decision‐making patterns regardless of whether decisions were made individually or collectively, while adults were more likely to choose “no action” in group decision‐making. Adolescents decreased their moral ratings in the group condition, while young adults believed that their changes to “no action” in groups were moral and changes to “action” were immoral. Finally, adult groups paid more attention to accountability about the scenario than other reasons. In conclusion, adolescents were more utilitarian when making decisions in moral dilemmas than young adults, who were less likely to make utilitarian choices when they are in groups.

## Conflicts of Interest

The authors declare no conflicts of interest.
